# LiB_13_: A New Member of Tetrahedral-Typed B_13_ Ligand Half-Surround Cluster

**DOI:** 10.1038/s41598-020-57769-2

**Published:** 2020-02-03

**Authors:** Hongxiao Shi, Xiaoyu Kuang, Cheng Lu

**Affiliations:** 1 Institute of Atomic and Molecular Physics, Sichuan University, Chengdu, 610065 China; 2 School of Mathematics and Physics, China University of Geosciences (Wuhan), Wuhan, 430074 China

**Keywords:** Macromolecules and clusters, Density functional theory

## Abstract

It will get entirely unusual derivatives with gratifying chemical bonding schemes for boron clusters by doping with lithium, the lightest alkalis. The geometric structures and electronic properties of the LiB_*n*_^0/−^ (*n* = 10−20) clusters have been studied through Crystal structure AnaLYsis by Particle Swarm Optimization (CALYPSO) structural search approach along with the density functional theory (DFT) calculations. The low-lying candidates of LiB_*n*_^0/−^ (*n* = 10–20) are reoptimized at the B3LYP functional in conjunction with 6–311 + G(d) basis set. Three forms of geometric configurations are identified for the ground-state structures of LiB_*n*_^0/−^ clusters: half-sandwich-type, quasi-planar and drum-type structures. The photoelectron spectra (PES) of the LiB_*n*_^−^ clusters have been calculated through time-dependent density functional theory (TD-DFT). A promising LiB_13_ with tetrahedral-typed B_13_ ligand half-surround cluster and robust stability is uncovered. The molecular orbital and adaptive natural density partitioning (AdNDP) analysis show that B-B bonds in the B_13_ moiety combined with the interaction between the B_13_ shell and Li atom stabilize the *C*_2*v*_ LiB_13_ cluster. Our results advance the fundamental understanding about the alkali metal doped boron clusters.

## Introduction

It is a significant milestone in the nanomaterials science that Kroto *et al*. discovered the C_60_ fullerene^1^, which aroused a surge of research activities about carbon. As the neighbor of carbon, boron has aroused much research^2–8^ because of its potential application and electron deficiency^9,10^. B_40_^11^, the boron analogue of C_60_, has also been experimentally observed^12^. Researches over the past few years manifested that the lowest-energy structures of the B_*n*_^−/0/+^ clusters tend to be planar\quasi-planar. Detailly, the ground-state structures of the B_*n*_^−^ clusters keep to be planar\quasi-planar till *n* = 36 (B_36_^−^)^13–15^, *n* = 20 for the B_*n*_ clusters^16^ and *n* = 16 for the B_*n*_^+^ clusters^17^. Soon after, the evolution from planar to fullerene-like structures, B_39_^−^^18^, for the B_*n*_^−^ clusters appeared when *n* approximately equal to 40. For the positive one, the cage-like structure which can be seen as a new borospherene contains 39 boron atoms^19^.

It will open a new chapter for boron clusters by doping with metal atom, which can get entirely unusual derivatives with gratifying chemical bonding schemes. In the last few years, there were great deals of reports about transition-metal-doped (TM-doped) boron clusters. These studies indicate that the configurations of the TM-doped boron clusters are contrast to that of pure boron clusters. TM-centered boron rings, Co@B_8_^−^, V@B_9_^−^, Nb@B_9_^−^, Ta@B_9_^−^, Ru@B_9_^−^, Ta@B_10_^−^^20–22^, have been experimentally observed. Subsequently, the Co-centered drum-type structure of CoB_16_^−^ cluster^23^ and the half-sandwich-type structure of Co/Ru/IrB_12_^−^ cluster^24,25^ have been found by Wang and coworkers. In addition, RhB_18_^−^ cluster^26^ has been found that there are two different configurations for the ground state and both of the structures have been observed experimentally. In the year of 2017, a unique tubular molecular rotor has been observed for the global minimum of the TaB_20_^−^ cluster^27^. The MnB_*n*_^−/0/+^ (*n* = 10−20) clusters have been systematically studied to reveal the geometric constructions of middle-sized boron clusters after doping of a manganese atom^28^. Not only the transition metals, other metallic elements also have been used as dopants. The Al_2_^+^[B_7_^3−^] and Al^+^[B_8_^2−^]^29^ clusters, both of which are umbrella-type structures, have been observed through experimental and theoretical studies. PrB_7_^−^, a rare-earth-atom-doped boron cluster, has been researched through photoelectron spectroscopy^30^. Doping of alkali metal atom into B_3_^−^ cluster, which can get stable AM^+^[B_3_^−^] (AM = Li-Cs) cluster, has been theoretically predicted^31^. In addition, Alexandrova and coworkers^32^ found a stable *C*_2*v*_ LiB_6_^−^ cluster using the *ab initio* study. There is also a theoretical studies of the small-sized LiB_*n*_ (*n* = 1–8) cluster^33^.

Although there are some studies about the Li-doped boron clusters, all the previous investigations are almost small-sized clusters except for LiB_12_^34^ and LiB_20_^35^. It is of crucial importance for us to have a systematical study of middle-sized Li-doped boron clusters. Here, extensive structure searches of the LiB_*n*_ cluster were carried out using the CALYPSO structural search method. Subsequently, the predicted structures were reoptimized through the DFT calculations. Furthermore, we discussed the inherent stabilities of the lowest-energy structures of the LiB_*n*_ clusters. Moreover, the theoretical PES of the ground-state anionic structures had been simulated. To interpret the stabilization mechanism, chemical bonding analyses were carried out.

## Results and Discussions

### Geometrical structures

**Figure 1:**
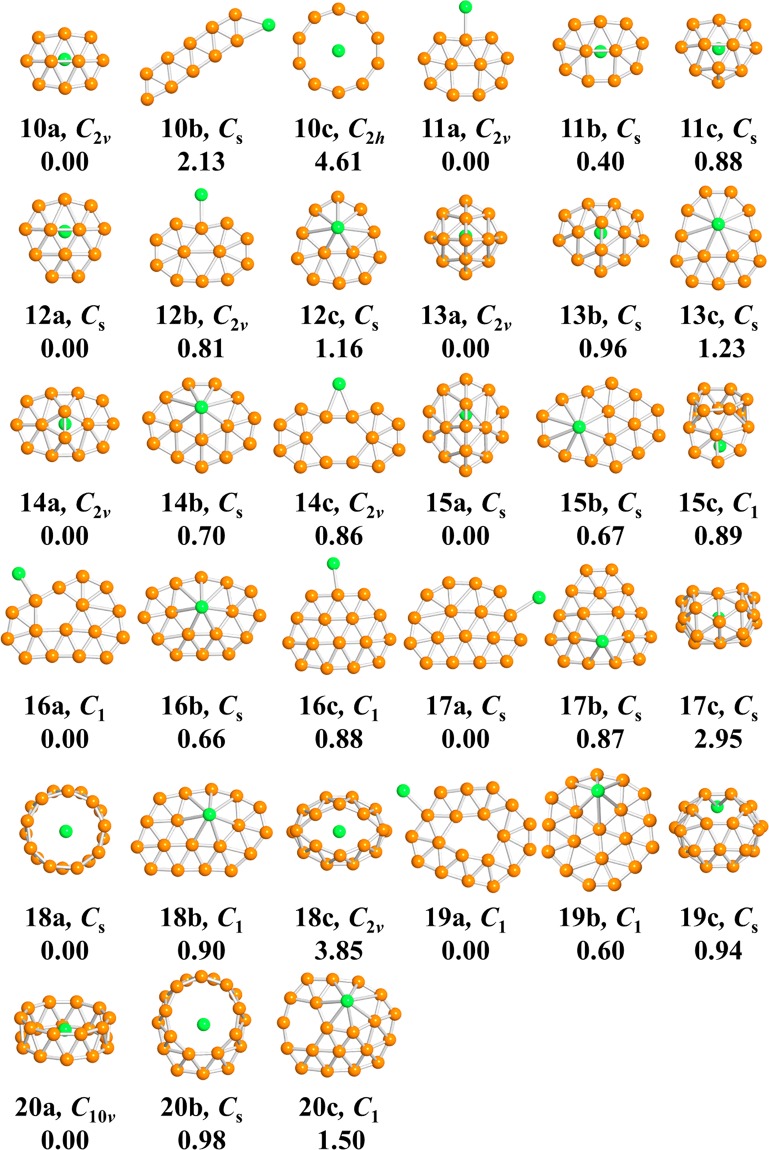
Optimized structures for LiB_*n*_ clusters. “na”, “nb” and “nc” indicates structures alphabetically. Point group symmetry along with the relative energy (eV) is also labeled under the structures.

**Figure 2:**
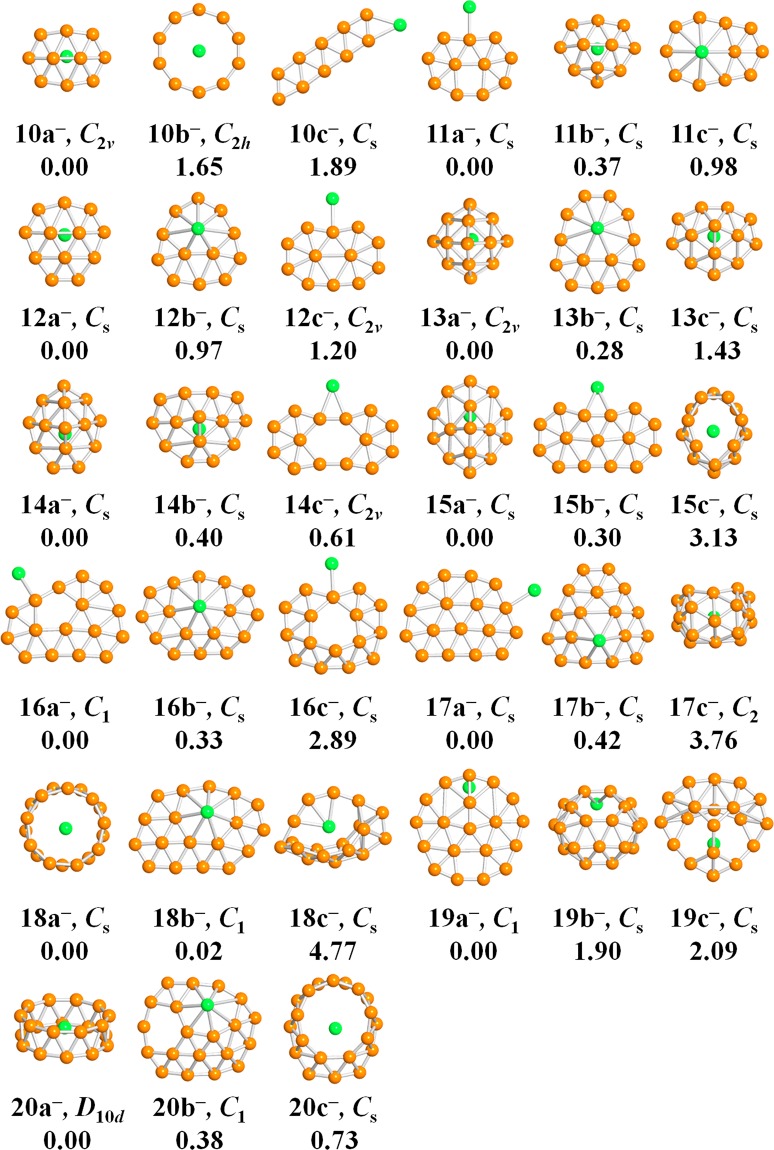
Optimized structures for LiB_*n*_^−^ clusters. “na^−^”, “nb^−^” and “nc^−^” indicates structures alphabetically. Point group symmetry along with the relative energy (eV) is also labeled under the structures.

The calculated low-lying isomers of neutral and anionic LiB_*n*_ (*n* = 10–20) clusters are exhibited in Figs. 1 and 2, respectively. Each isomers of the cluster is denoted by the labels: *n*a^/−^, *n*b^/−^ or *n*c^/−^. In brief, *n* is the number of B atoms of the corresponding cluster; a, b, c indicates the ground-state structure and the metastable structure, alphabetically. Beside the label, there is also the point group symmetry and relative energy (eV) of the corresponding structure. (The corresponding lateral views of the low-lying isomers are shown in Figs. S1 and S2 in the Supplementary Information.) As presented in Figs. 1 and 2, most of the structures, when *n* ≤ 15, grasp high point group symmetry. The lowest-energy structures of the LiB_*n*_^0/−^ (10 ≤ *n* ≤ 15) clusters present in forms of half-sandwich type, while for *n* ≥ 16 turn out to be quasi-planar and drum-type structures. Both the anionic and neutral ground-state structures of LiB_10_–LiB_15_, except for LiB_11_, are presented as half-surrounded structures, where the B_*n*_ moiety half surround to the Li atom. For the a/a^−^ structure of the LiB_10_ cluster, the B_10_ shell is a quasi-plan with two inner B atoms surround by an outer B_8_ ring. As exceptions, the structures of LiB_11_^0/−^ are plane structures. For the rest half-sandwich-type structures, the B_*n*_ fragments of 12a/a^−^ and 14a are more flat than that of 13a/a^−^, 14a^−^ and15a/a^−^. It should be noteworthy that the B_13_ ligand of the 13a/a^−^ is no longer planar or quasi-planar but a tetrahedral-typed structure, which is in stark contrast to that of pure B_13_ cluster^36^ through doping of a Li atom. All of the 16a/a^−^, 17a/a^−^ and 19a have a pattern that a quasi-planar B_*n*_ moiety with a doped Li atom connected to it, showing a quasi-planar structure. 19a^−^ possesses an intermediate structure of quasi-planar and half-sandwich-type. Both the 20a and 20a^−^ are beautiful drum-type structures. However, there are some distinctions between the structure of 18a/a^−^ and 20a/a^−^ where the Li atom is outside of the B_18_ drum. It is worth noting that the acquisition of an electron has little influence on the lowest-energy structures of LiB_*n*_^0/−^ clusters except for LiB_13_ and LiB_19_. Diversely, there are some changes for the low-lying isomers due to the acquisition of an electron.

### Photoelectron spectra

**Figure 3:**
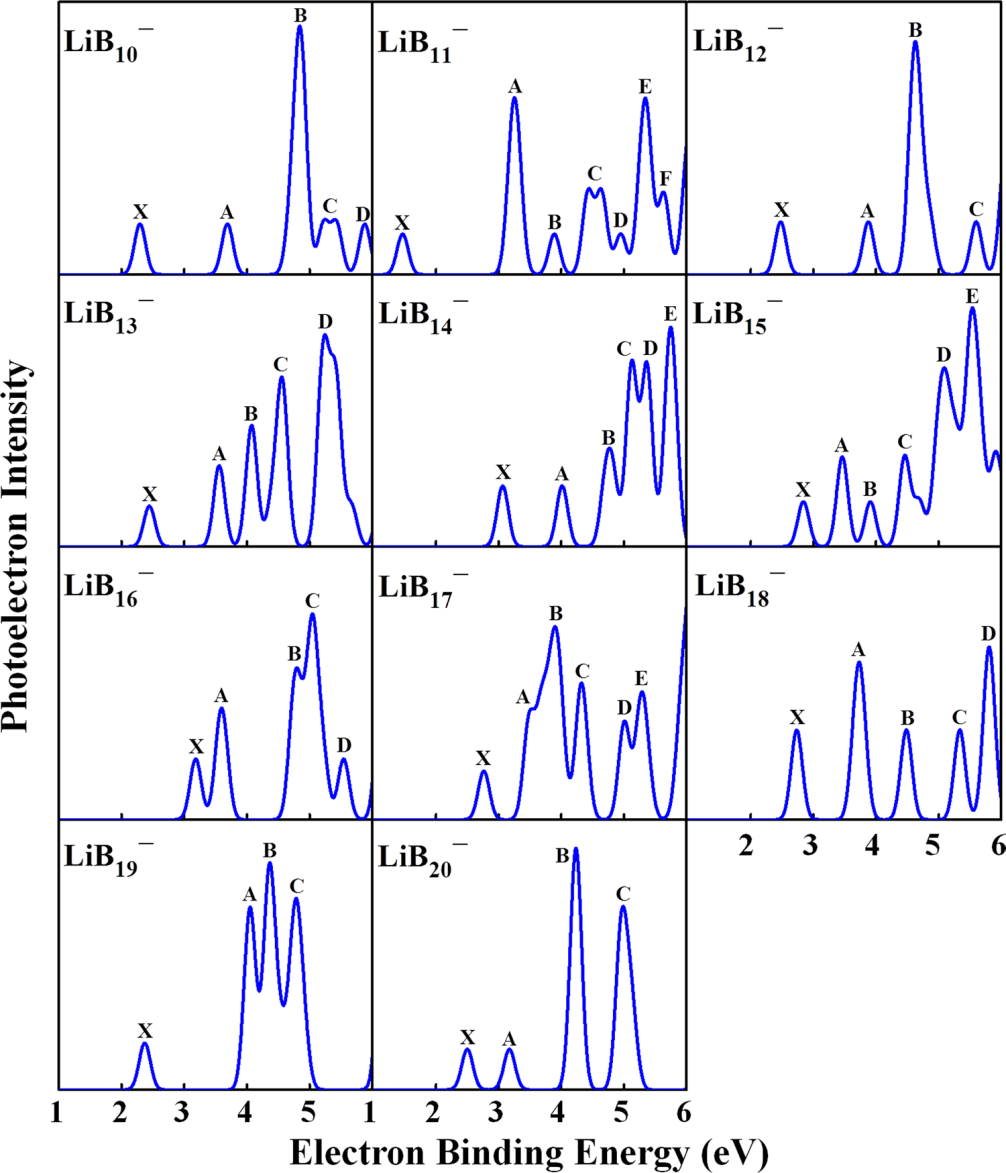
Calculated PES of the lowest-energy LiB_*n*_^−^ (10–20) clusters.

To deeply understanding the electronic structures of LiB_*n*_ cluster, the simulated PES of LiB_*n*_^−^ (*n* = 10–20) clusters are displayed in Fig. 3. The calculated PES together with the laboratorial PES of LiB_6_^−^ cluster^32^, a smaller species than our LiB_*n*_ cluster, are displayed in Fig. S3 in the Supplementary Information. For each spectrum, the location of the first peak (X peak) indicates the vertical detachment energy (VDE), which denotes electronic detachment transition from the ground-state anion to the corresponding ground-state or excited-state neutrals. The VDE values of the LiB_*n*_^−^ clusters are presented in Table S1 in Supplementary Information. The calculated spectrum of LiB_10_^−^ is sparse and the VDE is about 2.29 eV. Inversely, the spectrum of LiB_11_^−^ expresses a compact spectral pattern except for the X peak, which located at 1.47 eV. There are four major peaks of the LiB_12_^−^ PES and the VDE is about 2.48 eV, which corresponding to the position of the first peak. For LiB_13_^−^, there are five peaks and the VDE value is 2.44 eV. For the theoretical spectrum of LiB_14_^−^, the X Peak and A peak are very similar in shape and the X peak is located about 3.06 eV. The spectral pattern of LiB_15_^−^ PES is crowded, of which the VDE value is 2.84 eV. There are five peaks of the LiB_16_^−^ PES, of which the VED is 3.18 eV. The PES of LiB_17_^−^ is compact and the value of VDE is about 2.76 eV. There are five peaks of the PES of LiB_18_^−^ and the first peak is located at 2.73 eV. The VDE of LiB_19_^−^ is 2.37 eV and the X peak is far from the other three peaks. There are four sharp peaks in the sparse PES of LiB_20_^−^, of which the VDE value is 2.50 eV.

### Relative stabilities

In our recent work, the inherent stabilities of the LiB_*n*_^0/−^ clusters are determined by three criteria: average binding energy (E_*b*_), second-order differences of energy (Δ^2^E) and the energy gap (E_*gap*_). First, the E_*b*_ of LiB_*n*_^0/−^ clusters are calculated according to the following formulas:

1 $${E}_{b}(Li{B}_{n})=[nE(B)+E(Li)-E(Li{B}_{n})]/(n+1)$$

2 $${E}_{b}(Li{B}_{n}^{-})=[nE(B)+E(L{i}^{-})-E(Li{B}_{n}^{-})]/(n+1)$$

**Figure 4:**
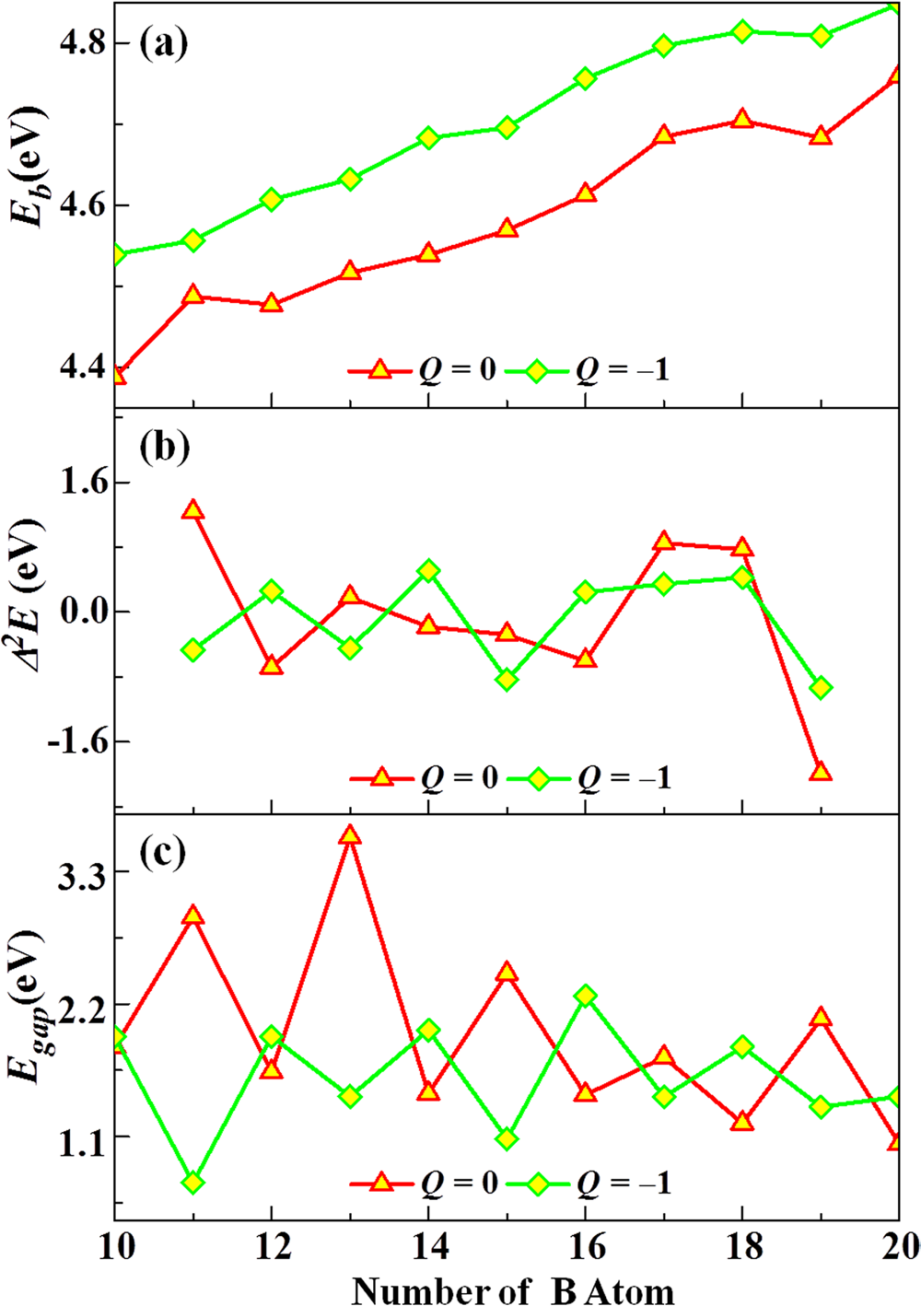
The (**a**) averaged binding energies (*E*_*b*_, eV), (**b**) second-order energy differences (Δ^2^*E*, eV) and (**c**) HOMO–LUMO energy gaps (*E*_*gap*_, eV) of the ground-state LiB_*n*_^*Q*^ (*n = *10–20, *Q* = 0, −1) clusters.

Among these, E express the energy of the matching atom or cluster. The values of E_*b*_ of the ground-state clusters are presented in Table 1 and visualized in Fig. 4(a). Larger E_*b*_ value is the representation of a stronger chemical stability. The anions LiB_*n*_ clusters are more inert than the neutral ones for that the neutral E_*b*_ curve sits below the anionic curve. It is obviously that both the anionic and neutral curves roughly increase with augmentation of the cluster size *n*, indicating that it is easier for the formation of the larger cluster. It is worth noting that, there is a distinct heave in the neutral curve when *n* = 11.

**Table 1:** The calculated electronic states, symmetries, *E*_*b*_ (eV) and *E*_*gap*_ (eV) of ground-state LiB_*n*_^0/−^ clusters.

	LiB_*n*_				LiB_*n*_^−^			
*n*	Sta.	Sym.	E_*b*_	E_*gap*_	Sta.	Sym.	E_*b*_	E_*gap*_
10	^2^A_2_	*C*_2*v*_	4.39	1.84	^1^A_1_	*C*_2*v*_	4.54	1.93
11	^1^A_1_	*C*_2*v*_	4.49	2.92	^2^A^”^	*C*_*s*_	4.56	0.72
12	^2^A′	*C*_*s*_	4.48	1.63	^1^A^'^	*C*_*s*_	4.61	1.93
13	^1^A_1_	*C*_2*v*_	4.52	3.58	^2^A_1_	*C*_2*v*_	4.63	1.43
14	^2^A_2_	*C*_2*v*_	4.54	1.46	^1^A′	*C*_*s*_	4.68	1.98
15	^1^A′	*C*_*s*_	4.57	2.45	^2^A′	*C*_*s*_	4.70	1.08
16	^2^A	*C*_1_	4.61	1.45	^1^A	*C*_1_	4.76	2.27
17	^1^A′	*C*_*s*_	4.68	1.76	^2^A^”^	*C*_*s*_	4.80	1.43
18	^2^A^”^	*C*_*s*_	4.70	1.21	^1^A′	*C*_*s*_	4.81	1.84
19	^1^A	*C*_1_	4.68	2.07	^2^A	*C*_1_	4.81	1.35
20	^2^A	*C*_10*v*_	4.76	1.04	^1^A	*D*_10*d*_	4.85	1.43

As a sensitive argument to reflect the relative stability, the Δ^2^E of the lowest-energy LiB_*n*_^0/−^ clusters is defined as:

3 $${\Delta }^{2}E(Li{B}_{n})=E(Li{B}_{n-1})+E(Li{B}_{n+1})-2E(Li{B}_{n})$$

4 $${\Delta }^{2}E(Li{B}_{n}^{-})=E(Li{B}_{n-1}^{-})+E(Li{B}_{n+1}^{-})-2E(Li{B}_{n}^{-})$$

Both the neutral and anionic Δ^2^E values can be fined in Fig. 4(b). Fig. 4(b) presents that the LiB_11_, LiB_13_, and LiB_17_ clusters along with the LiB_12_^−^, LiB_14_^−^, LiB_16_^−^ and LiB_18_^−^ clusters possess obviously higher Δ^2^E values which indicating their enhanced stability relative to the immediate neighbors.

The E_*gap*_ between the highest occupied molecular orbital (HOMO) and the lowest unoccupied molecular orbital (LUMO) is also a signature of the stability of the corresponding cluster. The higher E_*gap*_ values the stronger chemical stability of the given cluster. The calculated E_*gap*_ values of the lowest-energy LiB_*n*_^0/−^ clusters are presented in Table 1 and Fig. 4(c). It is distinct to notice that both of the E_*gap*_ curves express the even-odd oscillation behavior. There are two obvious outliers for LiB_11_ and LiB_13_ cluster suggesting that they are more inert than the others. Accordingly, we can determine that, from the results of E_b_, Δ^2^E, and E_*gap*_ above, the neutral LiB_11_ and LiB_13_ cluster can be viewed as “magic” clusters which are chemically inert.

### Chemical bonding analysis

**Figure 5:**
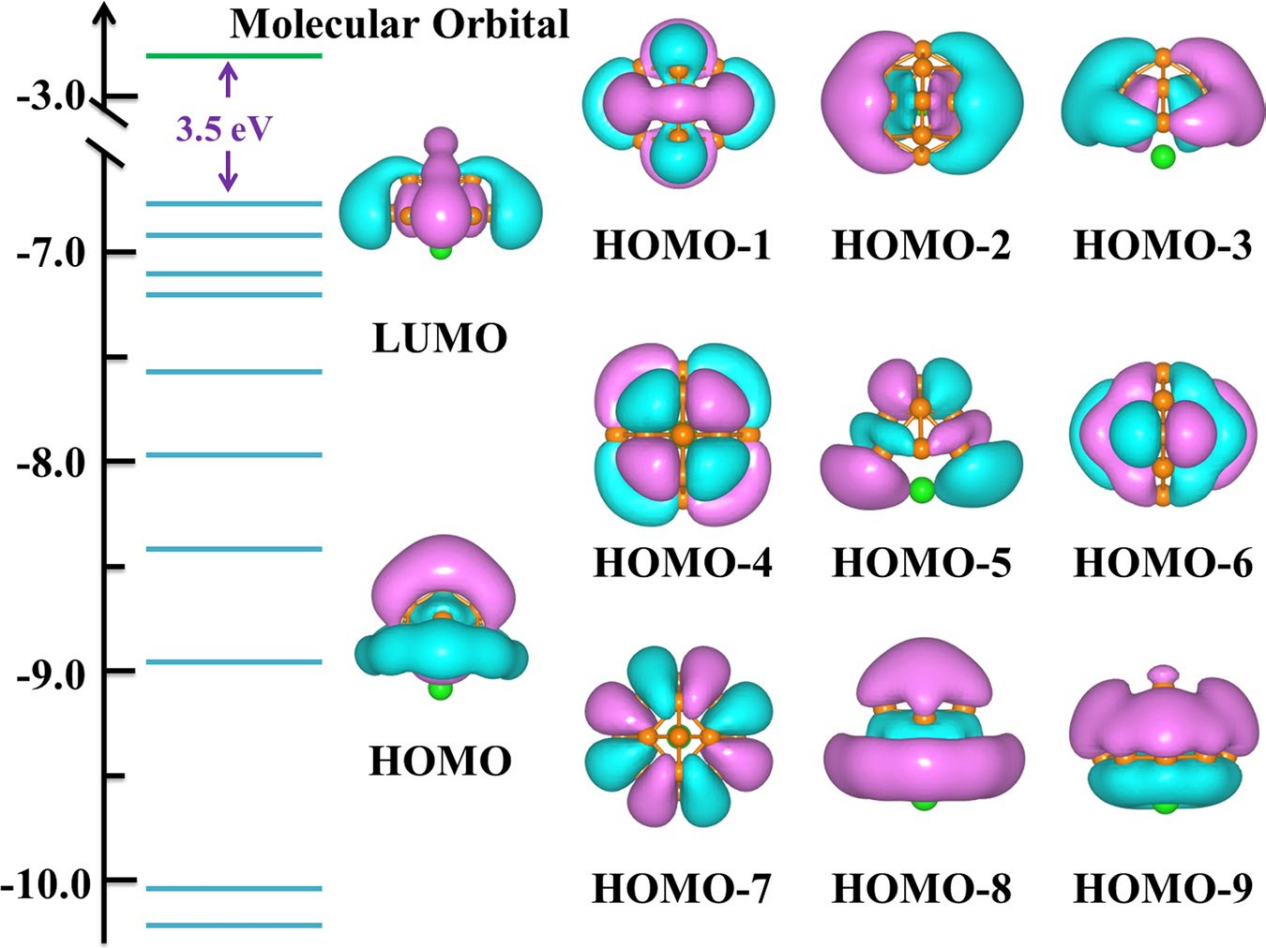
Visualized results of Molecular orbital and one-electron energy levels of the ground-state LiB_13_ cluster.

The molecular orbital (MO) and AdNDP analyses are presented to fully grasp the bonding mechanism of lithium-doped boron clusters. LiB_13_ (a half-sandwich-type structure, *C*_2*v*_) is chosen as a representation for its chemical stability and unusual geometric structure. There are eleven pictorial MOs near the HOMO of LiB_13_ along with their corresponding energy levels in Fig. 5. The LUMO of LiB_13_ cluster is a σ-hybrid orbital and primarily formed of 2p atomic orbital (AO) of boron atom. For the rest MO of LiB_13_, the nondegenerate HOMO, HOMO-8 and HOMO-9 orbital, which formed by p-type AO of B atom and s-type AO of Li atom, are σ orbital. However, as σ orbital, HOMO-1 is predominantly composed by B p-orbital of the B_13_ atoms moiety. The HOMO-a (a = 2–7) feature π orbital are primarily *p*-type AOs of the B atom. Based on the analyses, it has been found that the 2p AOs of boron atoms have made necessary contributions for these molecular orbital. Meanwhile, stabilize of the LiB_13_ structure is attributed to the mutual effect between the Li atom and the B_13_ fragment.

**Figure 6:**
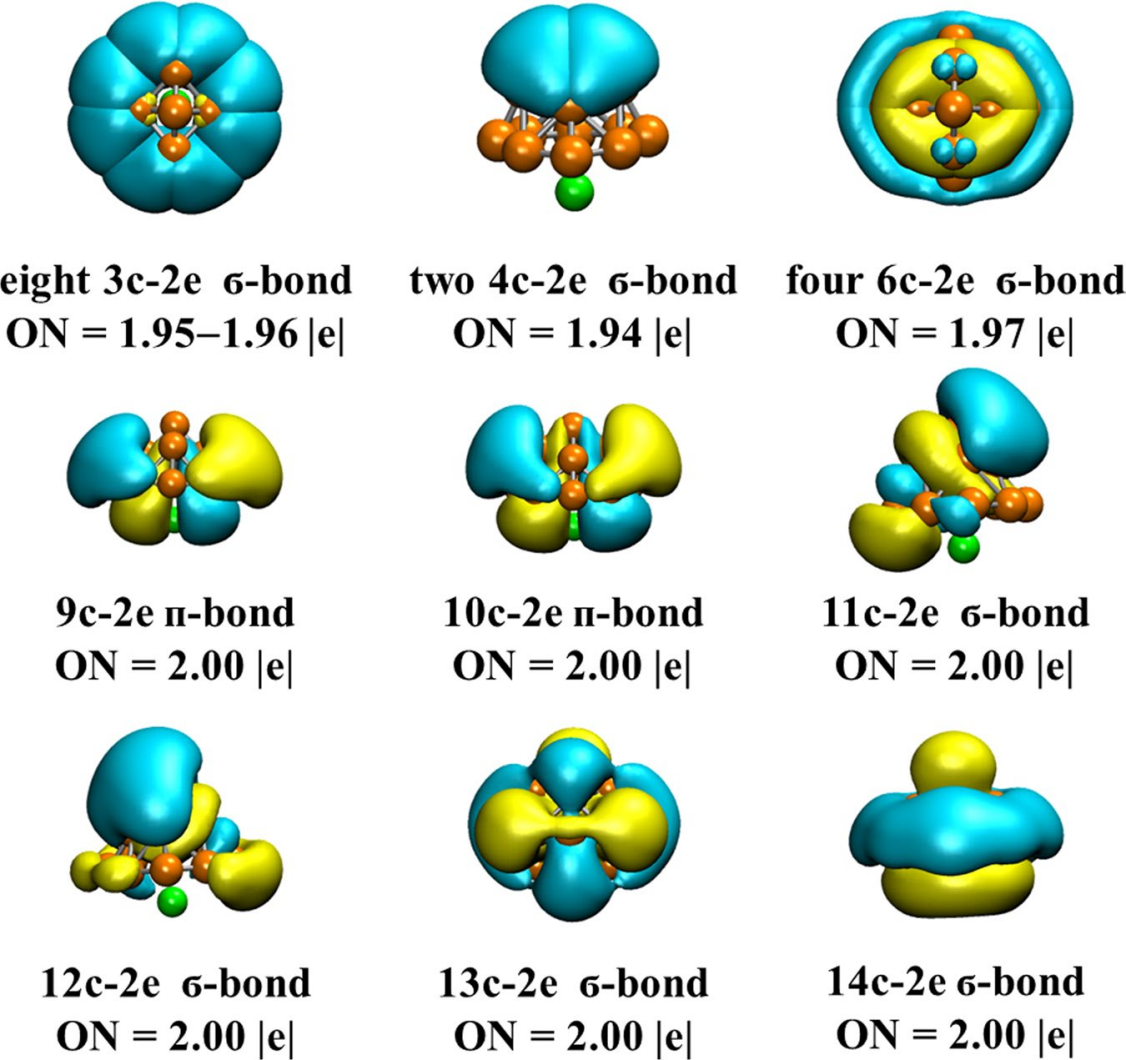
AdNDP (adaptive natural density partitioning) of the LiB_13_ cluster along with the ON (the occupation number).

To have a further understanding of the bonding properties of LiB_13_ cluster, the chemical bonding analysis by the means of AdNDP code was made. AdNDP algorithm describe the chemical bonding in terms of *n*c−2e (1 ≤ *n* ≤ the maximum number of atoms), which are obtained by partitioning the valence electron density matrix. The visualized results of LiB_13_ cluster together with their occupation number (ON) are presented in Fig. 6. All of the ONs approximately equal to the ideals values which lending credence to the AdNDP results we obtained. We divided these bonds into two categories: one work for the partial stability in the B_13_ shell and the other provide overall stability between the B_13_ moiety and the impurity atom Li. First, there are seventeen bonds on the B_13_ moiety and all of them are σ bond. The interactions of the B atoms in the peripheral B_8_ ring and the middle B_4_ ring are visualized by twelve σ bonds (eight 3c–2e bonds with ON = 1.95–1.96|e| and four 6c–2e bonds with ON = 1.97|e|). Meanwhile, the two 4c–2e σ bonds (ON = 1.94|e|) reveal the interactions between apical B atom and the four B atoms in the middle. Among the rest bonds, 11c–2e σ bonds, 12c–2e σ bonds and 13c–2e σ bonds, all the occupation numbers are 2.00|e|, also describe the B-B interaction in the B_13_ fragment. Then, there are three bonds for the integrity stability between the Li atom and the B_13_ shell and all of the three ON are 2.00|e|. Both of the 9c–2e π bonds and 10c–2e π bonds, formed by the 2 s orbital of Li atom the 2p orbital of B atoms, visualize the π mutual effect between the Li atom and part of the B_13_ shell. The connection between the entire B_13_ fragment and the Li atom is presented by the delocalized 14c−2e σ bond. Overall, both of the two groups of bonds earn substantial stabilization for the LiB_13_ cluster.

## Conclusion

As a conclusion, we have presented a systematical research of the neutral and anionic lithium-doped boron clusters through CALYPSO structural search approach and DFT calculations. The lowest-energy geometric structures of LiB_*n*_^0/−^ (*n* = 10–20) clusters are determined. The evolution of the lowest-energy structures is that: half-surrounded structures for size *n* from 10 to 15 and quasi-planar or drum-type structures for the larger species (*n* ≥ 16). The inherent stabilities have been analyzed by the average binding energy, second-order differences of energy and HOMO–LUMO energy gaps. A new tetrahedral-typed B_13_ ligand half-surround LiB_13_ cluster with high stability is identified. The molecular orbital and AdNDP analysis of the neutral LiB_13_ cluster suggest that B-B σ bonds in the B_13_ fragment combined with the strong interaction between B_13_ shell and Li atom stabilize the *C*_2*v*_ LiB_13_ cluster. This finding may provide guidance to future synthesis of boron-based nanomaterials.

## Computational Details

Our structural search of neutral and anionic lithium-doped boron clusters are implemented by utilizing CALYPSO code^37–40^. The capability of this method has been successfully confirmed in structural prediction of several systems with only given the chemical composition^41–45^. We have followed fifty generations to achieve the global-minimum structures during the structural predictions. Every generation contains thirty structures. Thus, we can obtain about 1500 isomers for each cluster size *n*. The top fifty low-lying candidates are selected and re-optimized using the all-electron DFT theory via the Gaussian 09 package^46^. The optimization calculations are performed using the B3LYP functional^47,48^ with 6–311 + G (d) basis set^49^, which is chosen for both of the B and the Li atom. Multiple spin states up to sextet and quintet are fully involved for the structure optimization. Vibration frequencies calculations are performed to ensure all the isomers are global-minimum. The PES of the ground-state LiB_*n*_^−^ clusters are calculated by means of the TD-DFT^50^. To further understanding the bonding mechanism of Li-doped boron clusters, the molecular orbital and AdNDP analyses^51^ are calculated utilizing the Multiwfn 3.3.8 program package^52^.

## Supplementary information

Supplementary Information.

## Data Availability

The data in this manuscript is availability.
