# Supplementary material for: LiB13: A New Member of Tetrahedral-Typed B13 Ligand Half-Surround Cluster
Source: Sci Rep. 2020 Feb 3;10:1642. doi: 10.1038/s41598-020-57769-2 (PMC6997382; doi:10.1038/s41598-020-57769-2)

Supplementary Information

LiB_{13}: A New Member of Tetrahedral-Typed B_{13} Ligand Half-Surround Cluster

Hongxiao Shi[1], Xiaoyu Kuang[1,*], Cheng Lu[2,*]

[1] Institute of Atomic and Molecular Physics, Sichuan University, Chengdu 610065, China

[2] School of Mathematics and Physics, China University of Geosciences (Wuhan), Wuhan 430074, China

[*]Correspondence author. E-mail: scu_kuang@163.com (Xiaoyu Kuang), and lucheng@calypso.cn (Cheng Lu)

Table S1. The calculated vertical detachment energy VDE (eV) of LiB_n^- clusters, along with the experimental data for comparison. [a] Ref. 32.

	VDE(eV)	
Cluster	Theo.	Exp.
LiB_{10}^-	2.29	--
LiB_{11}^-	1.47	--
LiB_{12}^-	2.48	--
LiB_{13}^-	2.44	--
LiB_{14}^-	3.06	--
LiB_{15}^-	2.84	--
LiB_{16}^-	3.18	--
LiB_{17}^-	2.76	--
LiB_{18}^-	2.73	--
LiB_{19}^-	2.37	--
LiB_{20}^-	2.50	--
LiB_6^-	2.37	2.57[a]

Figure S1. The side elevations of optimized structures of LiB_n clusters (n = 10–20).

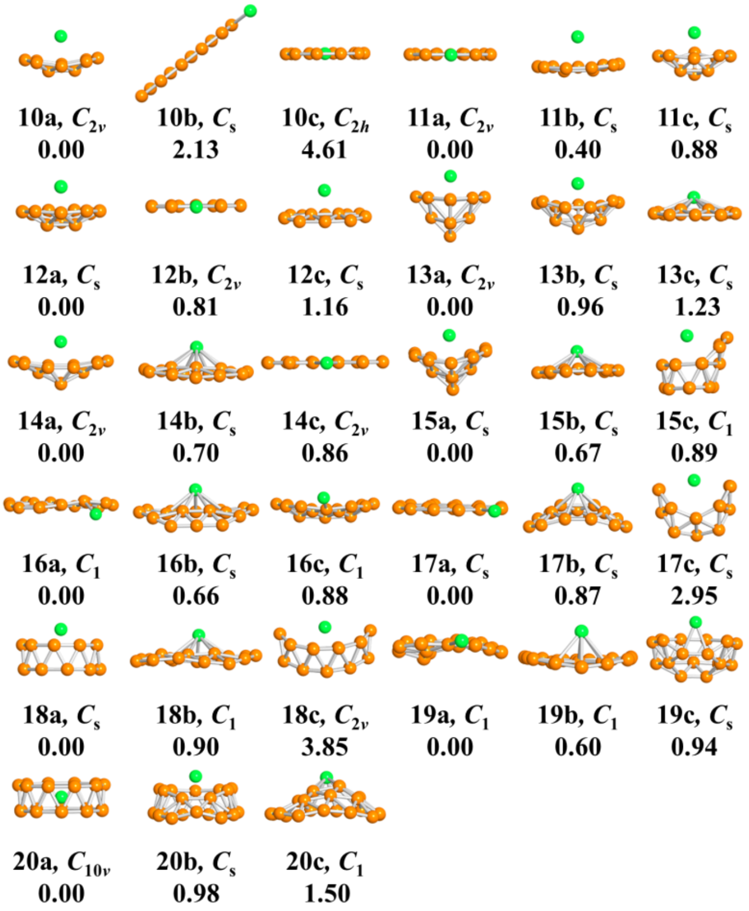

Figure S2. The side elevations of optimized structures of LiB_n^- clusters (n = 10–20).

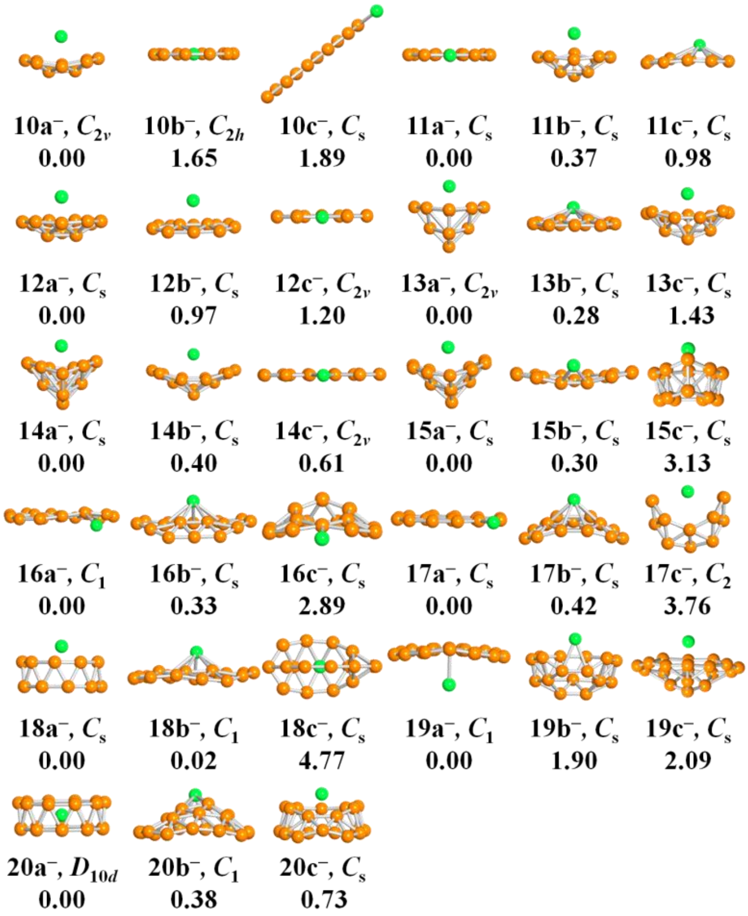

Figure S3. The simulated and experimental[32] PES of LiB_6^- clusters.

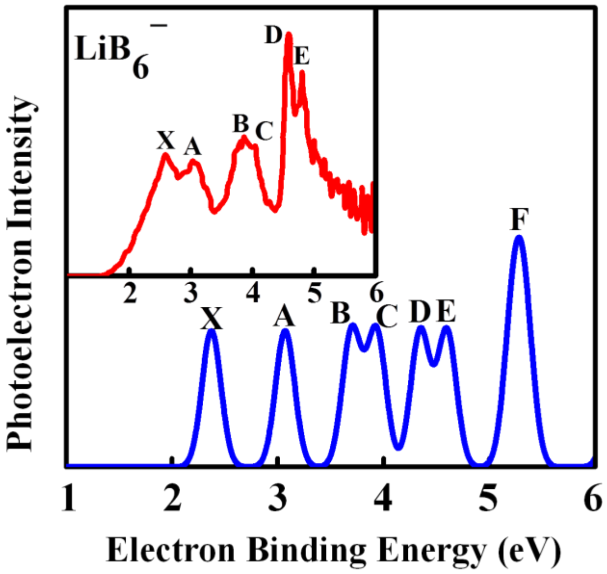

Supplement: Supplementary file 1 — Supplementary Information. [file 41598_2020_57769_MOESM1_ESM.pdf]
